# Qualification of Soybean Responses to Flooding Stress Using UAV-Based Imagery and Deep Learning

**DOI:** 10.34133/2021/9892570

**Published:** 2021-06-28

**Authors:** Jing Zhou, Huawei Mou, Jianfeng Zhou, Md Liakat Ali, Heng Ye, Pengyin Chen, Henry T. Nguyen

**Affiliations:** ^1^Division of Food Systems and Bioengineering, University of Missouri, Columbia, MO 65211, USA; ^2^Bioenergy and Environment Science & Technology Laboratory, College of Engineering, China Agricultural University, Beijing 100083, China; ^3^Fisher Delta Research Center, University of Missouri, Portageville, MO 63873, USA; ^4^Division of Plant Sciences, University of Missouri, Columbia, MO 65211, USA

## Abstract

Soybean is sensitive to flooding stress that may result in poor seed quality and significant yield reduction. Soybean production under flooding could be sustained by developing flood-tolerant cultivars through breeding programs. Conventionally, soybean tolerance to flooding in field conditions is evaluated by visually rating the shoot injury/damage due to flooding stress, which is labor-intensive and subjective to human error. Recent developments of field high-throughput phenotyping technology have shown great potential in measuring crop traits and detecting crop responses to abiotic and biotic stresses. The goal of this study was to investigate the potential in estimating flood-induced soybean injuries using UAV-based image features collected at different flight heights. The flooding injury score (FIS) of 724 soybean breeding plots was taken visually by breeders when soybean showed obvious injury symptoms. Aerial images were taken on the same day using a five-band multispectral and an infrared (IR) thermal camera at 20, 50, and 80 m above ground. Five image features, i.e., canopy temperature, normalized difference vegetation index, canopy area, width, and length, were extracted from the images at three flight heights. A deep learning model was used to classify the soybean breeding plots to five FIS ratings based on the extracted image features. Results show that the image features were significantly different at three flight heights. The best classification performance was obtained by the model developed using image features at 20 m with 0.9 for the five-level FIS. The results indicate that the proposed method is very promising in estimating FIS for soybean breeding.

## 1. Introduction

The climatic change increases the frequency of precipitations of higher magnitude. It is predicted a 30% increase in heavy precipitation events by 2030 [1]. Flooding is the second largest abiotic stress [2], causing approximately $3.75 billion economic loss in 2019 and $114 billion in total from 1995-2019 across the United States [3]. Flooding has caused a significant reduction in crop production, including soybean [2], rice [4], wheat [5], corn [6], and other crops. Flooding causes yield losses by reducing root growth, shoot growth, nodulation, nitrogen fixation, photosynthesis, biomass accumulation, stomatal conductance, and nutrient uptake [7]. Flooding is referred to as a water layer above the soil surface and can be classified into waterlogging (water-saturated soil with only the root system under anaerobic conditions) and submergence (all roots and a portion of or all shoots covered by water) [8]. Submergence is often seen in wetland crops (e.g., rice), while waterlogging is common in dryland crops including soybean and maize [7].

Soybean is an important legume crop widely used for human food, animal feed, biofuel production, and many other products owing to its high protein and edible oil content [7]. However, due to population growth, climate change, soil degradation, and pollution, the current arable land is decreasing, resulting in great pressure on the global food supply, including soybeans [9]. Soybean growth, productivity, and seed quality are adversely affected by flooding stress. Flooding can reduce soybean yield by 17 to 43% at the vegetative growth stage and 50 to 56% at the reproductive stage [2]. Waterlogging treatment for two days could reduce soybean yield by 27% [10].

In conventional soybean breeding, numbers of progenies are crossed by one parent from elite cultivars and another from naturally selected soybean species and evaluated under flooding environments. Soybean cultivars with flooding-tolerant trait and high-yielding potential can be identified based on visual observation by experienced breeders [11]. Visual rating was used to evaluate soybean injury caused by flooding stress in the field [12, 13]. However, visual-based evaluations are laborious and subjective to human bias and may not be sufficiently precise for breeding purposes [14]. Therefore, it is much needed to develop an efficient, effective, and unbiased tool to quantify flooding injuries under field conditions.

In recent years, UAV-based imaging technologies have been widely used in agriculture, including quantifying crop growth traits, such as plant height [15], canopy area [16], and leaf temperature [17], estimating yield [18], determining growth stages [19], and detecting plant stresses [20, 21]. Crops under flooding develop a series of morphological (e.g., increased plant height, leaf wilting, and decreased biomass) and physiological responses (e.g., closing of stomata, reduction of transpiration, and inhibition of photosynthesis) [8] that could potentially be detected and quantified by remote sensing approaches. Jiménez et al. [14] reported a significantly positive relationship (Pearson correlation *r* = 0.85 with *p* value < 0.001) between NDVI and visual evaluation rates of flooding damages on Brachiaria grasses and an estimated waterlogging tolerance coefficient (WTC, the ratio of plant shoot biomass under flooding stress to the one under control conditions) based on NDVI highly agreed (*r* = 0.7, *p* value < 0.001) with destructively measured WTC. Duarte et al. [22] detected significant differences in photosynthetic pigment contents and chlorophyll *a* fluorescence collected using a portable fluorometer between nonflooded and flooded Allophylus edulis tree plants. However, there are few studies reporting an effective method to estimate flooding injuries or classify tolerance abilities.

Therefore, the goal of this study was to investigate the potential of using UAV-based imagery in estimating soybean shoot injuries due to flooding stress under field conditions. There were three supportive objectives: (1) to evaluate the differences in image features among soybean genotypes with different shoot injuries, (2) to evaluate the impact of flight heights and image resolution on the effectiveness of image features in differentiating flooding injuries, and (3) to evaluate the classification performance of a deep learning model with UAV-based image features as predictors.

## 2. Materials and Methods

### 2.1. Experiment Field and Experiment Setup

**Figure 1:**
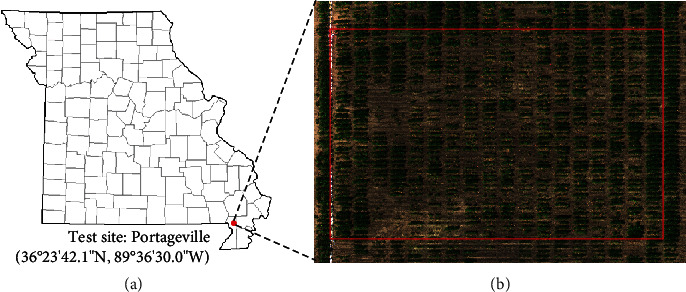
The field experiment. (a) The test site location. (b) Illustration of the experimental region in the field.

**Figure 2:**
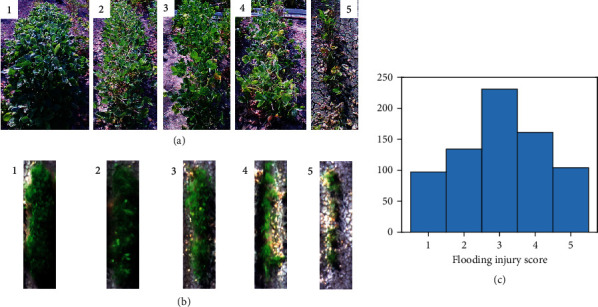
Representative images of soybean plots of different flooding injury scores (FISs). (a) Images were taken using a consumer-grade camera show example soybean plots at 1-5 level of FISs. (b) Soybean plots of different FISs show differently at the UAV images composing of the red, green, and blue channels from the multispectral images. (c) Histogram of the visually observed FISs for 724 soybean plots.

A field experiment (Figure 1) was conducted at the Lee Farm, Portageville, MO (36°23′42.1^″^N 89°36′30.0^″^W). A group of 382 soybean genotypes (maturity group IV to VI) was planted in single-row plots with two replicates on May 29, 2019, leading to a total of 764 soybean single-row plots. The soybean plots were planted with 2.13 m in length and 0.75 m spacing between rows. Waterlogging treatments were imposed by flood irrigation following previously described protocols [2, 12, 23]. Water was pumped on the field when 80% of the breeding plots were at the R1 growth stage [24]. The floodwater was raised to 5 to 10 cm above the soil surface and kept at this level for 8 days, and water was drained from the field when the injury was observed on checks that are sensitive to flooding stress. About 10 days after draining the water, each plot was rated using a five-level flooding injury score (FIS) based on the evaluation criteria described by Nguyen et al. [12], where “1” indicates no apparent injury and “5” indicates most plants severely injured or dead (Figures 2(a) and 2(b)). Excluding the plots with poor germination or completely dead before treatment, there were 724 plots with FIS recorded, and the distribution of soybean breeding plots of different FISs is shown in Figure 2(c).

### 2.2. UAV Imagery Data Acquisition

Two types of aerial images were collected simultaneously using a UAV imaging system. Multispectral images were acquired using a multispectral camera RedEdge-M (MicaSense, Seattle, WA, USA) that has a resolution (number of total pixels) of 1260 × 960 pixels. The multispectral camera was configured to take time-lapse images at 1 frame per second (fps). A GPS receiver was attached to the camera to provide geo-referencing information for each image frame as a part of metadata. All images with the EXIF (Exchangeable Image File Format) metadata were saved to an onboard SD card of the camera. Before and after each flight, a calibration reflectance panel (CRP) was imaged by holding the camera at about 1 m above the CRP and looking vertically in an open area (to avoid shadow) [19, 25].

Thermal images were collected using an infrared (IR) thermal camera (8640P, Infrared Cameras Inc., Beaumont, TX, USA) that has a resolution of 640 × 512 and an accuracy of ±1°C with the spectral band range of 7-14 *μ*m. The thermal camera was controlled using a single chip computer (Raspberry Pi 3B) to log thermal images to its onboard SD card at 1 fps. Prior to the flight, the camera was powered on for at least 15 min to warm up the sensor to acquire stabilized readings. The radiometric values of the thermal camera were factory calibrated, and a calibration file provided by the manufacturer was included in Raspberry Pi to correct the readings of each pixel.

Two cameras and their accessories (a power bank and a Raspberry Pi) were mounted on a UAV (DJI Matrice 600 Pro, DJI, Shenzhen, China) with the cameras facing the ground vertically (nadir view) during data collection. The UAV platform was controlled using a flight control App Autopilot (Hangar Technology, Austin, TX, USA). Images were acquired at 4 pm local time on Sept. 6, 2019, a clear day with an average air temperature of 33.4°C, relative humidity of 52%, and wind speed of 3 m/s. Images were taken at 20 m above ground level (AGL) at the speed of 7 km/h following a zigzag path to cover the field with the forward overlap ≥70% and side overlap ≥65%. Immediately after the completion of the mission at 20 m, the UAV platform was raised to 50 m and 80 m AGL to take images of the whole field. The ground sampling distances (GSDs) of the two types of UAV imagery at three flight heights are shown in Table 1.

### 2.3. Image Processing

The multispectral images collected at 20 m were processed using a UAV image processing software Pix4D Mapper (Pix4D, Lausanne, Switzerland) to generate orthomosaic images. Images and associated GPS information were read automatically from the EXIF metadata. The “Ag Multispectral processing” template was applied for this processing. A radiometric calibration procedure was conducted before processing to convert its image pixel numbers to reflectance values. Under the calibration option window, the image of the CRP panel in each band of the five bands was allowed to be selected and cropped, with the corresponding known reflectance values provided for each band [26]. An orthomosaic image of the IR thermal images at 20 m was generated using Agisoft PhotoScan Pro (Agisoft LLC, St. Petersburg, Russia) following the procedure described in a previous study [27]. Three parameters were set as “High” with Generic and Reference preselection for image alignment, “High” for reconstruction parameter, and “Moderate” for filtering mode. The orthomosaic images of the five-band multispectral camera as well as the IR thermal camera were all generated and exported as *.tif* images and then processed using the Image Processing Toolbox and Computer Vision System Toolbox of MATLAB (ver. 2016b, The MathWorks, Natick, MA, USA). The multispectral raw images collected at 50 and 80 m were directly processed without generating orthomosaic images due to the small number of images. To compare the NDVI variations at different flight heights, pixel numbers of individual raw images were converted into reflectance values using the same procedure with the orthomosaic images, referred as the unified-factor method. Raw pixel numbers were first converted to a common unit matric radiance (W/m^2/nm/sr) accounting for image biases, vignette effects, imager calibrations, and image gain. A unified scale factor between radiance and reflectance (0-1) was then calculated by dividing the radiance values of the panel image with its known reflectance in each band. Then, the reflectance of each raw image was obtained by multiplying its radiance values and the scale factor [28]. To avoid the assumption of consistent light conditions in the unified-factor method, pixel numbers at 50 and 80 m were rescaled in each band between 0 and 1 using the “*rescale*” function in MATLAB with the “*InputMin*” of 0 and “*InputMax*” of 65535 (2^16^-1), referred as the max-min method. Twenty points were randomly selected from each of the images taken at four corners, i.e., bottom left, top left, bottom right, and top right, to compare the difference of the same points screened at two different images in reflectance values converted using the max-min method.

**Figure 3:**
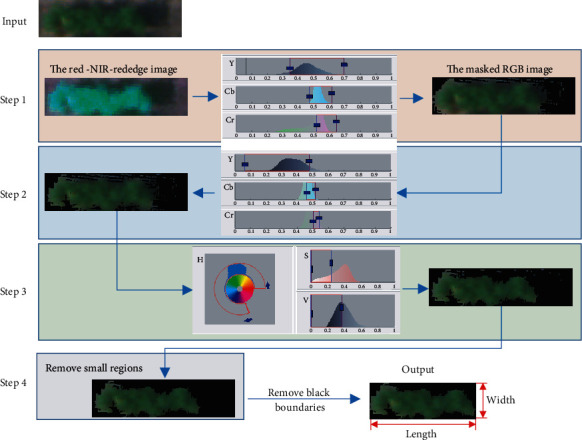
A stepwise procedure to remove the background of the multispectral images.

Individual soybean plots were separated from all the six single-channel orthomosaic images at 20 m by manually cropping a rectangle region of interest (ROI) around each plot. The ROI size varied to cover each soybean plot according to its canopy width and length. Overlapping between adjacent plots was avoided based on visual examination. Background (soil, shadow, and plant residues) was removed from the multispectral images with the assistance of the Color Thresholder app in MATLAB. After loading an image to the app, the image was represented in four color spaces: RGB, HSV [29], YCbCr [30], and CIE Lab [31]. In any selected color spaces, the color regions of the background in the space can be identified by drawing multiple freehand regions on the image. The color regions were then saved in a MATLAB script and applied to the images of other soybean plots. The background was removed for individual plots following a stepwise procedure (Figure 3) with one step focusing on removing one segment in the background. A three-layer image with the red, near-infrared (NIR), and red edge bands was loaded to the app in the first step, and color regions in the YCbCr space were selected to remove the majority of soil background from the image. In step 2, the remaining soil background close to the soybean canopy was removed from the masked RGB image using the YCbCr space. In step 3, shadows and fallen leaves that were in dark colors were removed using the HSV color space. In the last step, image regions were measured using the “*regionprops*” function with the “area” property in MATLAB, and those with an area less than 1% of the image were considered as noises and removed. The background of the thermal images was removed from individual plots using an unsupervised machine learning method *K*-means clustering [27].

Five image features were extracted for each soybean plot from the processed multispectral and IR thermal images, as representatives of canopy color (NDVI), shape (canopy area, canopy width, and length), and canopy temperature. As shown in Figure 3, the length and width of a soybean canopy were defined as the number of rows and columns of its multispectral images after the background was removed. Canopy area was defined as the total number of pixels in the multispectral images. NDVI was calculated using Eq. (1). Canopy temperature was calculated as the mean of pixel values of its thermal image.

(1) $$\begin{array}{c} \text{NDVI}=\frac{\text{NIR}-R}{\text{NIR}+R}, \end{array}$$

where NIR, *R*, and *G* are the reflectance values in each pixel of the NIR, red, and green bands of the multispectral images.

### 2.4. Data Analysis

Statistical analysis was performed in *R* studio (Version 1.1.414, RStudio, Boston, MA, USA). A one-way analysis of variance (ANOVA) with an honest significant difference (HSD) Tukey test was conducted to evaluate the differences in NDVI and temperature of each plot collected at different flight heights. In addition, the differences in canopy area, NDVI, and temperature among soybean plots with different FISs were also analyzed. ANOVA tests were conducted at a significance level of 5% using the “*aov*” function, and the Tukey tests were performed using the “*TukeyHSD*” function.

**Figure 4:**
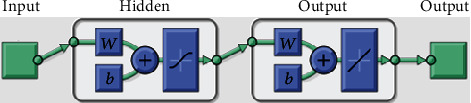
The architecture of the FNN model used to classify the FISs. *w* and *b* are trainable parameters that were iteratively optimized to minimize the model loss (error).

A feedforward neural network (FNN) model was developed to classify the FISs with the five image features as predictors. As one of the classic deep learning architectures, FNN models automatically learn from input data by tuning their trainable parameters hierarchically [32], and their variance-bias tradeoff for different datasets can be easily adjusted by controlling the numbers of neurons in the models. It has provided intelligent solutions to various agricultural applications, such as yield estimation [18, 33] and disease detection [34, 35]. As shown in Figure 4, a feedforward network consists of a series of layers. The first layer connects to the network input (five image features in this study), followed by a few hidden layers that create trainable parameters to be associated with the inputs. The final layer connects to the network's output (i.e., FISs in this study). The trainable parameters (*w* and *b*) will be iteratively optimized and updated to minimize model errors, according to specific optimization rules (i.e., training functions).

In this study, the FNN model was built using the “*feedforwardnet*” function in MATLAB with 10 neurons defined in the hidden layers. The function provides a structured FNN model and can be easily implemented by defining the number of hidden layers and training functions. The Levenberg-Marquardt optimization algorithm was selected as the training function that is considered the fastest backpropagation algorithm in the MATLAB Deep Learning toolbox and is highly recommended as a first-choice supervised algorithm [36, 37]. The model parameters and their settings used in this study were listed in Table 2. The outputs of the FNN model were categorized into five levels as shown in Table 3, each corresponding to one score of the five FISs. The 764 soybean lines were randomly split into training (90%) and testing (10%) datasets to train and validate the model during the training process. The performance was evaluated using the classification accuracy of the testing set as calculated in Eq. (2). In this study, all the computational analysis was performed on a desktop PC (Dell Optiplex 5050). The PC was configured as Intel (R) Core i7-7700 CPU (8 cores), 16 GB RAM memory, a 512 GB solid-state drive. The training and validating procedure for the FNN model was less than 2 min on the PC.

(2) $$\begin{array}{c} \text{Accuracy}=\frac{\text{No}.\text{of samples classified correctly in a test set}}{\text{Total No}.\text{of samples in a test set}}\times 100\%. \end{array}$$

## 3. Results and Discussions

### 3.1. Canopy Temperature at Three Flight Heights

**Figure 5:**
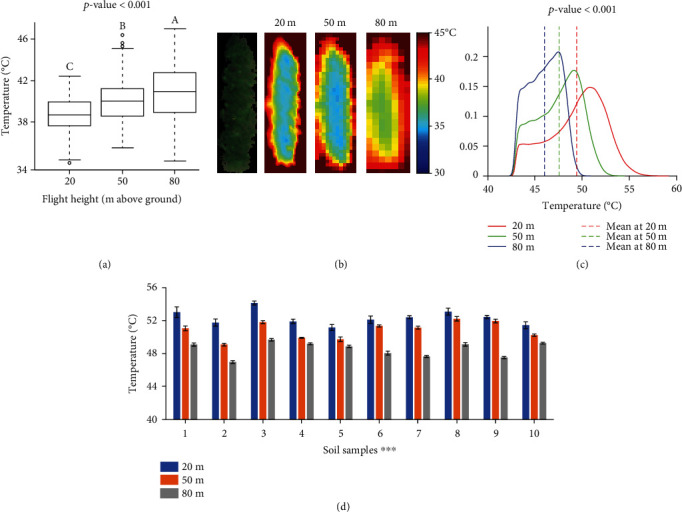
The comparisons of canopy temperature measured at three flight heights. (a) The boxplots of soybean canopy temperature were collected at three flight heights. The end of the boxes defines the 1^st^ and 3^rd^ quartile, with a plot at the median and error bars defining the 10^th^ and 90^th^ percentiles. The lowercase letters above bars indicate the significant difference among these means at a 0.05 significance level. (b) An RGB image of a soybean plot captured by the multispectral camera at 20 m and its thermal images at three heights. (c) Density plots of the temperature values of soil at three heights. (d) Column plots of the temperature values of ten soil samples that were manually cropped from the thermal images at three heights. ∗∗∗ indicates a significance level of *p* = 0.001.

Figure 5 shows the comparison of temperature values of soybean plots and background extracted from the IR thermal images collected at three flight heights. From Figure 5(a), a significant difference was observed among three groups of soybean canopy temperature taken at different flight heights. The average canopy temperature of all the soybean plots increased consistently from 39.0 to 40.3°C and 41.1°C when flight height increasing from 20 to 50 m and 80 m. However, it is noticed in Figure 5(b) that the temperature of the background significantly decreased from 49.4 to 47.6 and 46.0°C as the flight height increased. In addition, there were significant differences (*p* value < 0.001) in the average temperature values of ten selected locations of bare soil (Figure 5(d)) at three heights. The average temperature values of the ten soil samples were 52.3, 50.9, and 48.5°C at 20, 50, and 80 m, respectively. Even though there was no evidence that the variations of plant canopy temperature were caused by the fluctuations in radiation, air temperature, humidity, and wind speed during data collection, this contradictory pattern eliminated the possibility that the increase in temperature values of plant canopies was due to an environmental temperature surge.

Infrared thermography creates temperature images of an object by detecting infrared waves (7.5 to 14 *μ*m) emitted by an object and converting the radiation readings to temperature [38]. The variations in image temperature measurements due to shooting distance changes could be caused by combined effects of atmosphere composition, image resolution, and solar radiation [39]. The underestimation of soil temperature in higher flight might be due to the absorption of the infrared radiation (emitted by soil) by ambient gases and particles in the atmosphere [40]. It was confirmed by the observations of Ball et al. (2006) [41] that the temperature of emerging lava from a volcano vent decreased by 53°C by increasing the viewing distance from a thermal camera from 1.5 to 30 m and a further decrease of 75°C at a viewing distance of 250 m. Faye et al. [39] also reported a negative effect of shooting distance on surface temperature.

The increase of plant temperature with the increase of flight height might be because of the mixture effect of radiation energy from both plants and soil at a higher altitude. The temperature of bare soil was significantly higher than that of plants, which made the mixed temperature higher. Figure 5(d) shows an example image of a soybean plot captured at 20 m, and Figure 5(e) shows its thermal images at three heights. With the increase of flight height, more pixels of the image edge had a higher temperature that was close to soil. Image pixels were getting less sensitive to variances in soybean canopy and blurry at the boundaries between canopy and background. The average temperature increased due to the inclusion of pixels representing the background (hotter than plants).

In addition, the temperature measurements are subject to errors from atmospheric attenuation by atmospheric scattering caused by particulate material in the atmosphere [41]. Even though atmospheric attenuation corrections have been applied using internal correction functions in cameras, these corrections assume a uniform viewing distance across an image and take a mean relative humidity and air temperature from the frame into consideration. However, when the camera viewing over large distances, the viewing distance across an image varied due to camera movements and mounting errors, leading to possible varied temperature and relative humidity between the camera and the object under investigation over the image.

### 3.2. Canopy NDVI at Three Flight Heights

**Figure 6:**
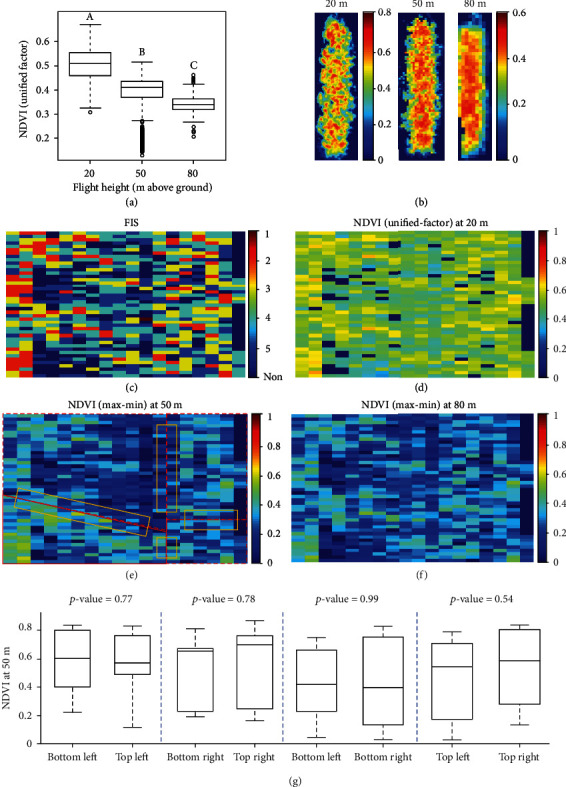
The comparisons of NDVI measured at three flight heights. (a) The boxplot of NDVI of all soybean plots collected at three flight heights. The end of the boxes defines the 1^st^ and 3^rd^ quartile, with a plot at the median and error bars defining the 10^th^ and 90^th^ percentiles. The lowercase letters above bars indicate the significant difference among these means at a 0.05 significance level. (b) The false-color images of an example plot (Figure 5(d)) in NDVI. (c) FIS map of the experiment field. Each grid represents the FIS of a single soybean line. (d–f) NDVI maps at 20, 50, and 80 m with each grid representing an averaged NDVI value of each plot. Soybean plots at 50 m were segmented from four images. The polygons in (e) mark the four images that the soybean plots were segmented from and the images were taken from the bottom left, top left, bottom right, and top right corner. (g) Four pairs of boxplots with each pair comparing the difference of ten points screened at two different corners. The rectangles in (e) display the regions that the points were randomly selected from.

Figure 6 shows the comparison of NDVI of all the soybean plots collected at three flight heights. In Figure 6(a), NDVI values were calculated with the reflectance values converted by the unified-factor method. There are significant differences observed among the three heights. From 20 to 50 m, the average NDVI values over all the soybean plots decreased from 0.51 to 0.37 and then decreased to 0.34 at 80 m. False-color images in NDVI of a soybean example plot in Figure 5(d) display similar patterns among three flight heights that higher NDVI values dominated and gathered in the center of the plot. However, NDVI images at 20 m had a larger range than the other two and captured more variances among leaves due to the higher pixel resolution. It was noticed that the boxplot at 50 m had a long tail with some low NDVI values that were from the image at the bottom left corner (Supplement Figure S1a). As the unified-factor method assumes consistent light conditions during each flight mission, a sudden light change happened possibly when the drone was over the corner but was not corrected in the conversion.

The difference among flight heights could be further investigated using NDVI maps for the experiment field. The map at 20 m shows the most distinguishable patterns for classifying FISs. The maps at 50 and 80 m have similar patterns with the one at 20 m but with fewer variations, which is consistent with the observations in Figures 6(a) and 6(b). The maps in Figures 6(e) and 6(f) were generated with each grid representing an averaged NDVI value of each plot that was calculated using the max-min method. As in Figure 6(g), no significant differences were observed in each of the four regions covered two images at different corners, indicating the effectiveness of the max-min method. As light changes compensated by adjusting camera exposure time during image formation (longer exposure time for darker imaging conditions), low NDVI values were not observed in the bottom left corner of Figure 6(e).

### 3.3. Relationships between Image Features and FIS

**Figure 7:**
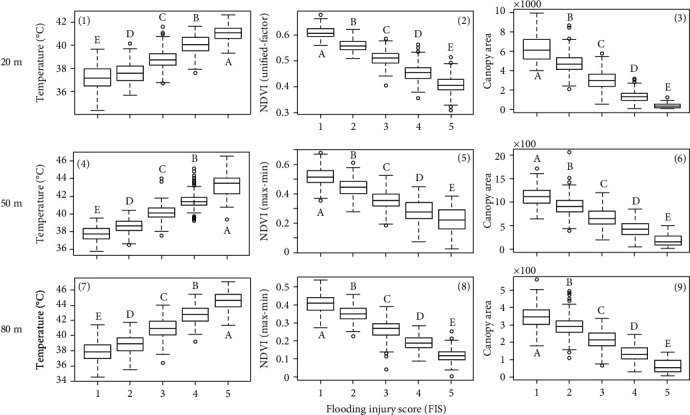
Relationships between three image features and FIS. (1)–(3) are the boxplots of temperature, NDVI, and canopy area at 20 m. (4)–(6) are those at 50 m. (7)–(9) are those at 80 m. The NDVI values at 50 and 80 m were calculated using the reflectance values converted by the max-min method. The lowercase letters above bars indicate the significant difference among these means at a 0.05 significance level.

The differences in each of the three image features among soybean plots of different FISs at three flight heights are shown in Figure 7. It can be seen that there are significant differences in the means of each image feature obtained from all three flight heights, respectively, indicating that the flight height had a limited effect on the ability of image features to distinguish the FIS of soybean plots due to flood stress.

#### 3.3.1. Canopy Temperature

It can be seen that at all three flight heights, the canopy temperature values were significantly different among the soybean plots with different FISs. Less injured genotypes had lower canopy temperature than the severely injured ones. One of the major causes of the difference in crop canopy temperature is leaf transpiration [42] during which the openings of leaf stomata allow the diffusion of carbon dioxide gas from the air for photosynthesis [43]. Transpiration cools plants. The transpiration rate is regulated by controlling the size of the stomatal apertures and is also influenced by evaporative demands [44]. Flood-sensitive plants reduce their water absorption [45, 46] as a consequence of the reduction of root hydraulic conductivity under flooding [8, 47, 48]. In response to flooding, plants decrease stomatal conductance and transpiration rate by stomatal closing to regulate the water balance of plants and prevent leaf dehydration [45], consequently leading to high leaf temperature.

Leaf canopy temperature is a proxy to assess variations in leaf transpiration rate caused by environmental treatments or genetic variations [42]. Hou et al. [49] estimated soybean transpiration rate under four irrigation treatments using canopy temperature acquired by a high-resolution hand-hold thermal imager, and the results strongly correlated with field-based transpiration measurements. Spatial variations of leaf transpiration rate could also be visualized with the assistance of image-based temperature measurements. Lapidot et al. [50] estimated the transpiration rates of five tree species using ground thermal images and compared them to the direct measurements of a gas exchange system. It was reported that leaf temperature and the measured transpiration rates were significantly and negatively related, and the estimated transpiration rates yielded low errors (mean absolute errors ranged between 0.99 to 1.51 mmol H_2_Om^−2^ s^−1^) with the measured ones.

Leaf canopy temperature has also been considered as a promising indicator of water/heat-related plant stresses. For example, Li et al. [51] evaluated canopy temperature and transpiration rates of two rice genotypes under heat stress, and the results show that the heat-tolerant genotype had significantly leaf higher transpiration rates as well as lower temperature. Zhou et al. [21] investigated the potential in classifying drought-tolerant and sensitive soybean genotypes using a machine learning method with canopy temperature and other image features as predictors. The drought-tolerant genotypes had significantly lower canopy temperature than the sensitive ones, and the model reached an average classification accuracy of 80%.

#### 3.3.2. NDVI and Canopy Area

It can be seen from Figures 7(b), 7(e), and 7(h) that there were significant differences among NDVI values of soybean plots with different FISs showing a trend that NDVI values decreased as FISs increased. The low NDVI values in flooding sensitive genotypes might be caused by their low leaf chlorophyll contents attributed by low photosynthesis [52, 53]. In the short term of flooding, plant photosynthesis decreases as a result of a restriction of CO_2_ uptake due to stomata closing [54–56]. If flooding continues in time (a week or so), a decrease in the photosynthetic capacity of mesophyll cells [53, 57] leads to a further reduction of photosynthesis.

The NDVI measures differences of reflectance in the red and near-infrared regions of the spectrum [14] and is widely used to quantify the plant phenotypic performance [21]. The leaves of a healthy plant absorb more red light and reflect more near-infrared light, resulting in higher NDVI values than plants under stress [58]. NDVI has been used to estimate leaf chlorophyll contents [58], photosynthetic activity [59, 60], plant biomass [61, 62], yield [63, 64], and responses to stresses, such as salt [65] and drought [21]. This study demonstrated a potential alternative for quantifying plant damage due to flood stress in field conditions.

The negative effects of flooding on photosynthesis lead to a low growth rate in flooded plants [8], and consequently early leaf senescence and reductions in leaf area [66]. In this study, a significant difference in canopy area is observed among soybean plots with different FISs (Figures 7(c), 7(f), and 7(i)). Though the decreased rates of canopy area were not quantified due to the lack of canopy area before flooding, our observation could be supported the reductions in leaf/canopy area induced by flooding found in other plant species, such as kenaf [67] and sorghum [68].

Based on the observation by Striker et al. [56], negative effects on both flood-tolerant and sensitive species caused by flooding did not occur until a week of flooding. However, when flooding was discontinued, the flood-tolerant genotypes recovered their stomatal behavior and transpiration rates similar to those under the control group. Therefore, crop features representing their postflooding recovery is more conclusive for assessments of its tolerance [8].

### 3.4. Classification of FISs Using the FNN Model with Canopy Temperature, NDVI, Area, Length, and Width

**Figure 8:**
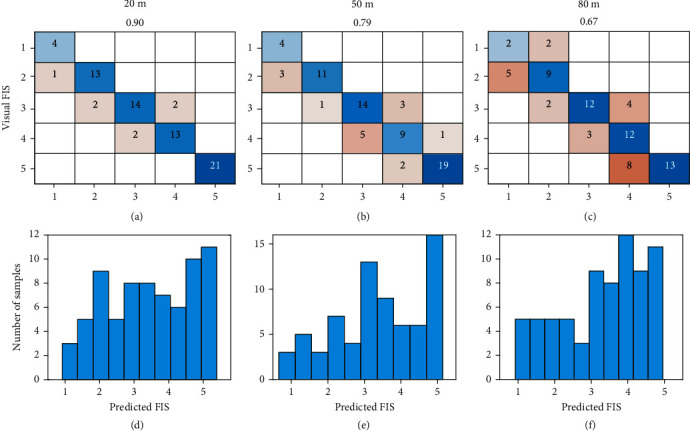
Classification performance of the FNN model with canopy temperature, NDVI, canopy area, length, and width collected at three flight heights. (a–c) are confusion tables of the five-level FIS. (b–f) are histograms of the predicted FIS in testing sets.

The classification accuracy on the testing set (72 samples) using the FNN model with the five image features collected at 20, 50, and 80 m is shown in Figure 8. From the confusion tables of the five-level FIS, the model at 20 m reached the highest accuracy (90%), followed by that at 50 m (79%) and 80 m (67%). The results confirmed the effects of flight heights on individual image features and also reflected a negative effect on the accuracy of the prediction of FIS. In 20 m, only one or two samples were misclassified between adjacent classes. As flight height increases, more samples were observed in wrong classes, especially for those who were damaged severely (class 3 to 5). However, no sample was classified to nonadjacent classes in all three heights, implying the ability of the image features to distinguish flooding injuries.

Figures 8(d)–8(f) show the histograms of the FNN outputs on the testing sets. The continuous predictions allow breeders to select any percentage of soybean plots. For example, eight samples (top 10%) or 15 samples (top 20%) of the testing set could be selected based on the predicted FIS. Among the top 10% ranks of these predictions, six level-1 plots and one level-2 plot were selected by the model at 20 m, eight level-1 plots by the 50 m model, and five level-1 and two level-2 plots by the 80 m model.

The selection of flight height lies in the trade-off between costs and accuracy. At the 20 m flight height, 637 five-band multispectral images and 497 IR thermal images were collected in 12 min to cover the whole field (0.22 ha), while four shots were taken at 50 m and only one shot at 80 to cover the field. During image preprocessing, it took over 2 h to stitch images at 20 m for each camera. Although the classification performance at 20 m outcompeted those at higher flight heights, errors could be introduced in other studies by potential light changes during data collection due to increasing screening time (not observed in this study) and the stitching procedures. Additionally, costs increased with the increase of collection time, such as labor costs and the need to invest more batteries and storage. Even though open-source algorithms are available, the most popular stitching applications require an expense to purchase licenses, such as Pix4D mapper, Agisoft PhotoScan Pro, and Sony Fast Field Analyzer.

Effective evaluation of germplasm and mapping populations for crop injuries under flooding conditions is essential to identify DNA elements (genes or markers) that underlie or are associated with flooding tolerance [7]. By marker-assisted selection and genomic selection in breeding programs, these stress-tolerance elements could be introduced into elite breeding lines to improve diversity and to support the sustainability of crop production during the current patterns of extreme climatic shifts [11]. Traditional visual evaluations of FIS are laborious often biased by the examiner and may not be sufficiently accurate [69]. Image-based features have been studied to quantify phenotypic traits of plant shoots and estimate flooding-induced injuries under field conditions [14]. However, none has been reported to estimate FIS effectively. Results from this study indicate the model performance at 50 m is acceptable in soybean breeding programs. Compared to previous studies, our study examined the efficiency of image-based canopy temperature, NDVI, and canopy area in differentiating FISs, explored the rationale behind these features, and proposed a deep learning algorithm to estimate FISs for practical use. The methods used in this study could be scaled up to other crops that are sensitive to flooding stress.

## 4. Conclusion and Future Study

The potential of estimating soybean plant injury caused by flooding stress using UAV-based image features under field conditions was investigated in this study. FISs of 724 soybean plots were taken using visual ratings when soybeans show flooding symptoms. Aerial images were taken when the soybean lines were scored using a five-band multispectral and an IR thermal camera at 20, 50, and 80 m above ground. Five image features, i.e., canopy temperature, NDVI, canopy area, width, and length, were extracted from the images at three flight heights. A deep learning model was proposed to classify FIS using the features. Image features showed significant differences at three flight heights and those collected at higher height were less powerful in explaining variations in target objects due to decreasing ground sampling distances. There was still a significant difference in each of the three image features among soybean plots with different FISs at three flight heights. The best classification performance was reached by the model at 20 m with 0.9 for the five-level FIS, followed by the model at 50 m with 0.79 and 0.67, respectively. The selection of flight height lies on the trade-off between costs and accuracy.

In this study, we investigated the concept that the UAV-based images and deep learning models can effectively detect soybean flooding responses to the flooding stress and accurately classify the FIS by visual observations. Yet, the proposed image processing pipeline is not fully automated to output tabulated image features for each line. The FNN model was trained by data collected in one environment and not yet ready for breeding practices. We will improve our pipeline with full automation and enhance the model using more data of genetic and environmental variations in the future. In addition, genetic analyses, e.g., genome-wide association studies (GWAS), will be conducted to identify functional gene loci regulating the flooding tolerance in soybeans that could be transferred into the current germplasm by marker-assisted selection.

## Figures and Tables

**Table 1:** Ground sampling distances (mm/pixel) and the total number of the multispectral and the IR thermal images at three flight heights.

Image type	Ground sampling distances (mm/pixel) and total image number		
	20 m	50 m	80 m
Multispectral	13.2, 637	34.7, 4	55.6, 1
IR thermal	23.4, 497	68.0, 4	108.8, 1

**Table 2:** FNN model parameters used to classify the FISs.

Parameters	Definition	Value
hiddenSizes	The number of hidden layers	10
trainFcn	Training function	trainlm
Epochs	Maximum number of epochs to train	1000
Goal	Performance goal	0
max_fail	Maximum validation failures	6
min_grad	Minimum performance gradient	1e-7
mu	Initial learning rate	0.001
mu_dec	Decrease factor for the learning rate	0.1
mu_inc	Increase factor for the learning rate	10
mu_max	Increase factor for the learning rate	1e10

**Table 3:** Categorization of the FNN outputs.

FNN output	Estimated FIS
[0, 1.5)	1
[1.5, 2.5)	2
[2.5, 3.5)	3
[3.5, 4.5)	4
[4.5, ∞)	5

## Data Availability

The data and programming codes are freely available upon request.

## Supplementary Materials

Supplementary Materials Figure S1: NDVI values at 50 and 80 m converted using the unified-factor method.
