# Supplementary figures and images for: Qualification of Soybean Responses to Flooding Stress Using UAV-Based Imagery and Deep Learning
Source: Plant Phenomics. 2021 Jun 28;2021:9892570. doi: 10.34133/2021/9892570 (PMC8261669; doi:10.34133/2021/9892570)

## Slide 1
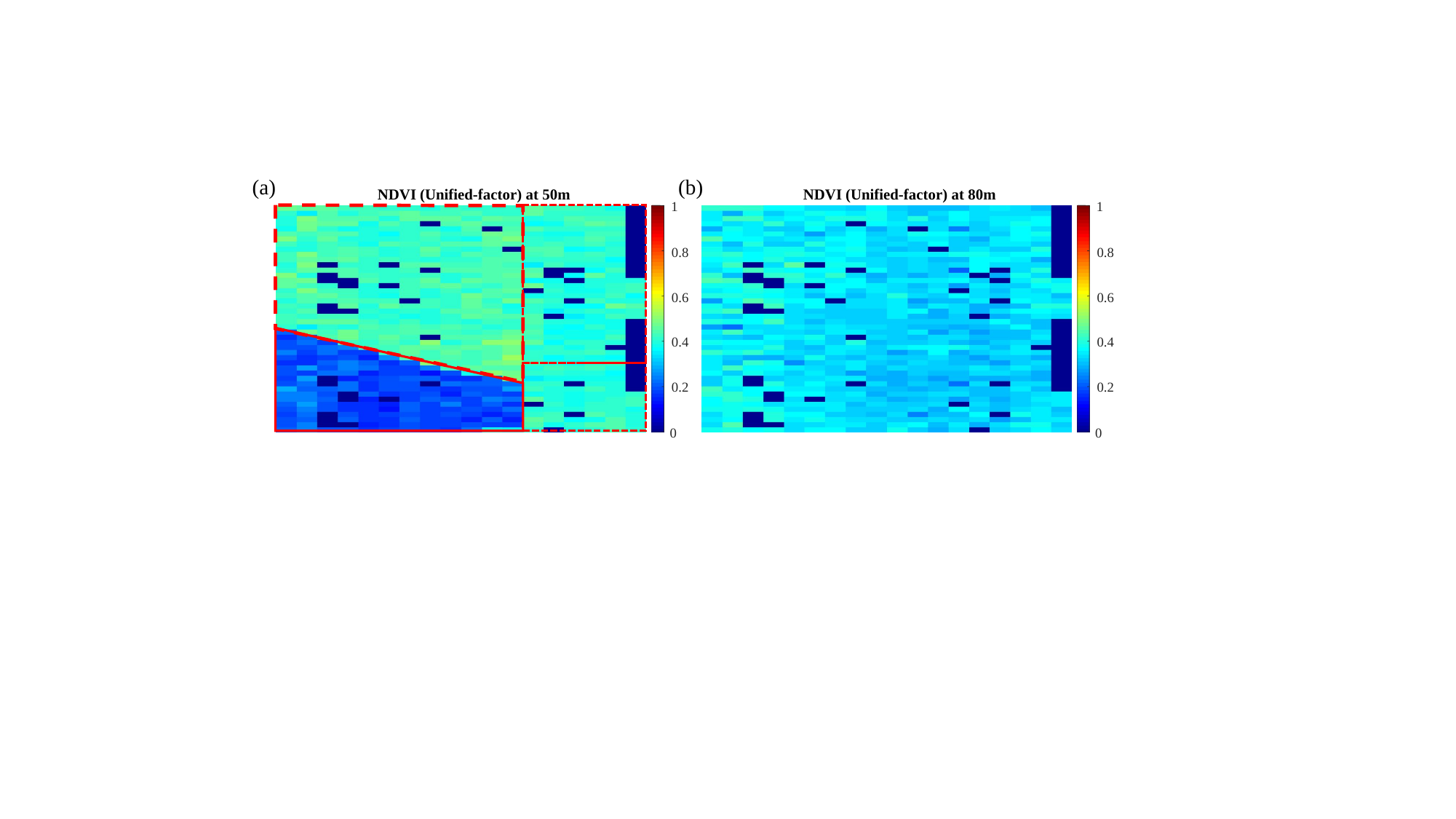

(a)
(b)
NDVI (Unified-factor) at 50m
NDVI (Unified-factor) at 80m
1
1
0.8
0.8
0.6
0.6
0.4
0.4
0.2
0.2
0
0

Supplement: Supplementary Materials — Figure S1: NDVI values at 50 and 80 m converted using the unified-factor method. [file 9892570.f1.zip › FigureS1.pptx]

Supplementary


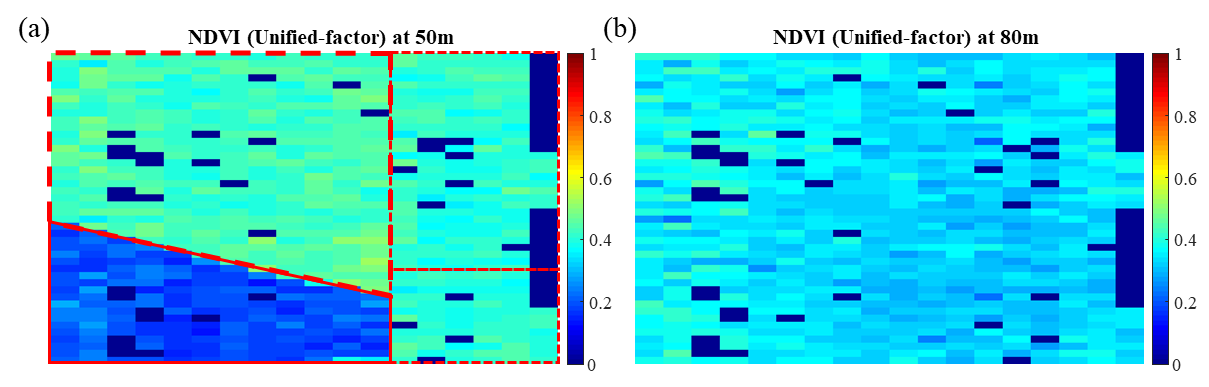


Fig. S1 NDVI values at 50 and 80 m converted using the unified-factor method

Supplement: Supplementary Materials — Figure S1: NDVI values at 50 and 80 m converted using the unified-factor method. [file 9892570.f1.zip › Supplementary.docx]
